# A tightly controlled gene induction system that contributes to the study of lethal gene function in *Streptococcus mutans*

**DOI:** 10.1080/20002297.2023.2253675

**Published:** 2023-09-07

**Authors:** Yongliang Li, Guanwen Li, Xuliang Deng

**Affiliations:** a Department of Cariology and Endodontology, Peking University School and Hospital of Stomatology, Beijing, People’s Republic of China; b National Engineering Laboratory for Digital and Material Technology of Stomatology, NMPA Key Laboratory for Dental Materials, Beijing Laboratory of Biomedical Materials, Peking University School and Hospital of Stomatology, Beijing, People’s Republic of China; c Department of Geriatric Dentistry, Peking University School and Hospital of Stomatology, Beijing, People’s Republic of China

**Keywords:** Gene induction system, xylose-induction, FtsZ, Streptococcus mutans, lethal gene function

## Abstract

Effective control of gene expression is crucial for understanding gene function in both eukaryotic and prokaryotic cells. While several inducible gene expression systems have been reported in *Streptococcus mutans*, a conditional pathogen that causes dental caries, the significant non-inducible basal expression in these systems seriously limits their utility, especially when studying lethal gene functions and molecular mechanisms. We introduce a tightly controlled xylose-inducible gene expression system, TC-Xyl, for *Streptococcus mutans*. Western blot results and fluorescence microscopy analysis indicate that TC-Xyl exhibits an extremely low non-inducible basal expression level and a sufficiently high expression level post-induction. Further, by constructing a mutation in which the only source FtsZ is under the control of TC-Xyl, we preliminarily explored the function of the *ftsz* gene. We found that FtsZ depletion is lethal to *Streptococcus mutans*, resulting in abnormal round cell shape and mini cell formation, suggesting FtsZ’s role in maintaining cell shape stability.

## Introduction

Dental caries is a widespread infection disease that can cause pain, tooth loss, and decreases the quality of life. Numerous studies have proven the etiological role of *Streptococcus mutans* (S. *mutans*) in dental caries [1]. *S. mutans* is a conditionally pathogenic bacterium that naturally resides in the human oral cavity. In addition to caries, *S*. *mutans* has been reported to cause infective endocarditis [2] and participate in cardiovascular diseases, including stroke [3]. Therefore, exploring the biology of this organism is important for developing treatments for dental caries and other *S. mutans*-associated diseases. The genome of *S. mutans* standard strain UA159 contains 1,963 open reading frames [4] (ORFs), 11% of these were reported as the essential genes [5], showing great potential as targets for antimicrobial drug development. Although the encoding products of these genes are necessary for cell growth and shape determination, the study of their function and mechanism remains technically challenging due to the difficulty of culturing gene knock out mutations.

Methods commonly used in other model organisms to study the function of a lethal gene mainly rely on a robust gene induction expression system that knocks out the endogenous gene while exogenously expressing a copy of that gene through a tightly controlled induction system. Currently, only a few gene-inducible expression systems have been proven available for *S. mutans*. TetR/TetO system uses tetracycline as the induction compound, and it has been proven to exhibit tight repression in *S. mutans* [6]. However, since tetracycline is a broad-spectrum antimicrobial agent, high concentration of tetracycline may affect the growth of *S. mutans*, limiting the amount of inducer and leading to low induction level in *S. mutans*. Previous studies have also reported inducible expression systems based on sugar metabolisms, such as sucrose inducible promoters from gene *scrB* [7], fructose-inducible promoters from gene *fruA* [8], and lactose-inducible system LacR/LacA [9]. Unfortunately, all these systems exhibit fairly strong levels of basal expression. In addition, although these induction compounds are non-toxic for *S. mutans*, they are readily metabolized sugars by *S. mutans*. As a result, they are not suitable for studies requiring strict control of carbon sources.

A previous study reported the xylose-based gene-inducible expression systems Xyl-S1 and Xyl-S2, which are most widely used in *S. mutans* [10]. Xyl-S1 has been reported to produce greater than 600-fold induction levels based on luciferase activity, whereas Xyl-S2 showed lower uninduced basal expression. Unlike the induction compounds mentioned above, xylose is non-toxic and unmetabolizable to *S. mutans*, making this system suitable for a variety of studies. However, we found these two systems still exhibited a high basal expression level. Therefore, there is still a need for an inducible gene expression system with both low basal expression levels, strong inducibility, and broad applicability in *S. mutans*. To meet this goal, here we constructed a tightly controlled xylose-inducible gene expression system (TC-Xyl), derived from Xyl-S1, and analyzed its basal expression and inducibility using Western blot and fluorescent microscopy. FtsZ is a core protein involved in cell division and is highly conserved in almost all bacteria. Deletion of *ftsZ* results in cell death, limiting the study of the function and molecular mechanism of FtsZ in *S. mutans*. Thus, to illustrate the specific advantages of TC-Xyl, we preliminarily investigated the function of FtsZ in *S. mutans.*

## Materials and methods

### Bacterial strains and culture conditions

The bacterial strains used in this study are listed in Table 1. All bacterial strains were cultured aerobically (95% air, 5% CO_2_) at 37°C. The chemically competent *Escherichia coli* cell Trans5α (Transgene tech) was grown in Luria-Bertani medium (LB) or LB agar plates supplemented with 50 μg/ml ampicillin. The *S. mutans* strains were cultured in Todd Hewitt Broth (TH) or on brain heart infusion (BHI) agar plates. For the natural transformation, *S. mutans* cells were grown in TH medium supplemented with 0.3% (wt/vol) yeast extraction. The antibiotic-resistant colonies were selected on the BHI plates supplemented with 1000 μg/ml spectinomycin or 10 μg/ml erythromycin. For the protein expression, D (+) xylose was added to the culture medium with different concentrations (wt/vol).

**Table 1.** Strains used in this study.

Strains	Genotype and description	Reference
UA159	*S. mutans* str UA159	Preserved in Central Laboratory, Peking University School and Hospital of Stomatology
pYL01:F-m	UA159; pYL01:FtsZ-mNeongreen, Str^R^	In this study
pZX9:F-m	UA159; pZX9:FtsZ-mNeongreen, Str^R^	In this study
YL001	UA159; ΔftsZ, pYL01:FtsZ-mNeongreen, Str^R^, Erm^R^	In this study
YL002	UA159; ΔftsZ, pZX9:FtsZ-mNeongreen, Str^R^, Erm^R^	In this study
Trans5α	Chemically Competent Cell	Transgene

### Construction of TC-Xyl cassette

**Figure 1.**
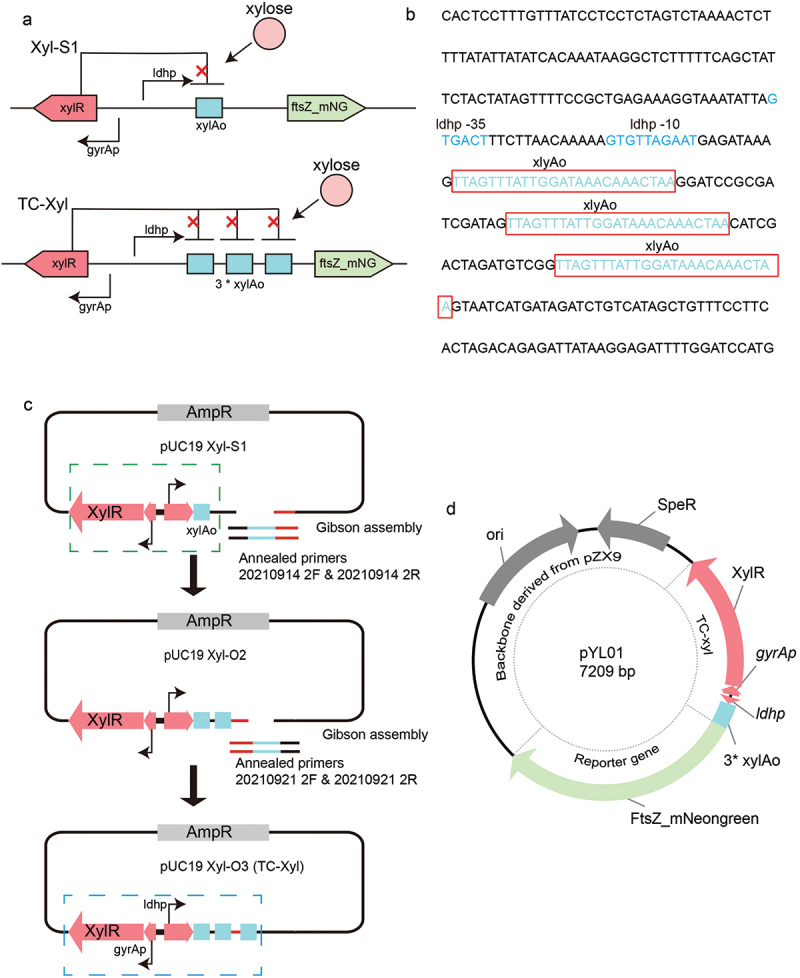
Schematic and sequence of the TC-Xyl. (a) Illustration of the genetic organization of induction elements in the TC-Xyl and Xyl-S1, the schematic diagram of Xyl-S1 was adapted from ref 10. (b) The sequence of the repetitive sequence of xylAo, the xylAo are marked in red box, promoter elements are shown in blue. (c) Displays the schema for TC-Xyl construction. Xyl-S1 and TC-Xyl are marked by green and blue dot line box, respectively. (d) The plasmid map of pYL01. The proportions in the schematic do not represent the actual proportions.

The primers used in this study are listed in Table 2. Primers 202109022F and 202109022R were used to amplify the *xylR/xylAo* expression cassette Xyl-S1 from plasmid pZX9 (gift from Zhoujie Xie); Primers 202109021F and 202109021R were used to amplify the linear plasmid pUC19 (pUC19). Following this, we assembled these two fragments via Gibson assembly kit (YEASEN, China) to get plasmid pUC19:Xyl-S1 (Figure 1(c)), and subsequently transformed into *E. coli* trans 5α and selected LB plates with 50 μg/ml ampicillin. Next, we used 202109141F and 202109141R to amplify linear pUC19:Xyl-S1. Primers 202109142F and 202109143R were mixed at equimolar concentration and annealed in ddH2O in a thermocycler, 95°C 5 min and then decreased by 5°C every 5 min until it reached room temperature. The annealed primers were cloned into pUC19:Xyl-S1 to get plasmid pUC19:Xyl-O2 via Gibson assembly kit. The plasmid pUC19:Xyl-O3 (three repetitive xylAo, also named TC-Xyl) was constructed by the same strategy with the primers 20210922 1f, 20210922 1r, 20210922 2F and 20210922 2R.

**Table 2.** Primers used in this study.

Function	Primer name	Sequence
pUC19:Xyl-S1	20210902 1F	CAAACTAAGGATCCGCGCGAAGATCTCGGCGTAATCATGGTCATAGCTG
	20210902 1R	AGAAGATCGATTTTCGTTCGTGAATAGAATTCACTGGCCGTCGTTTTAC
	20210902 2F	GTAAAACGACGGCCAGTGAATTCTATTCACGAACGAAAATCGATCTTC
	20210902 2R	ATTACGCCGAGATCTTCGCGCGGATCCTTAGTTTGTTTATCCAATAAACTAACTTTATC
pUC19:Xyl-O2	20210914 1F	CATCGACTAGATCTCGGCGTAATCATGGTC
	20210914 1R	CTATCGATCGCGGATCCTTAGTTTGTTTATCCAAT
	20210914 2F	GGATCCGCGATCGATAGTTAGTTTATTGGATAAACAAACTAA
	20210914 3R	TGATTACGCCGAGATCTAGTCGATGTTAGTTTGTTTATCCAATAAACTAA
pUC19:Xyl-O3	20210922 1f	GTAATCATGATAGATCTGTCATAGCTGTTTCCTGTGTG
	20210922 1r	CTAACCGACATCTAGTCGATG
	20210922 2F	CATCGACTAGATGTCGGTTAGTTTATTGGATAAACAAACTAAGTAATCATGATAGATCT
	20210922 2R	AGATCTATCATGATTACTTAGTTTGTTTATCCAATAAACTAACCGACATCTAGTCGATG
pYL01	20211021 4F	CTCTTGCCAGTCACGTTACGTTATTTATTCACGAACGAAAATCGATCTTC
	20211021 4R	CTCCTTATAATCTCTGTCTAGTGAAGGAAACAGCTATGACAGATCTATCATGATTAC
	20211021 5F (2)	CACTAGACAGAGATTATAAGGAGATTTTGGATCCATGGCATTTTCATTTGATGCAGCAT
	20211021 5R	GATGCCTGGCAGTTTATGGCGCGGCCGCTTATTACTTGTACAGCTCGTCCATGCCCAT
	20211021 6F	TAAGCGGCCGCGCCATAAACTGCCAGGCATC
	20211021 6R	AATAACGTAACGTGACTGGCAAGAG
EGFP qPCR	20230717 1F	GACAAGCAGAAGAACGGCATCAAG
	20230717 1R	TCACGAACTCCAGCAGGACCATG
16s qPCR	20210426 6F	GGCGACGATACATAGCCGACCT
	20210426 6R	CGTCCATTGCCGAAGATTCCCT
mNeongreen qPCR	20230717 2F	GACTGGTTTCCCTGCTGACGGT
	20230717 2R	CCATTGGCTTGGCAAAGGTGTAG

For the construction of plasmid pYL01, we used primers 20211021 4F and 20211021 4R to amplify the TC-Xyl expression cassette from plasmid pUC19:Xyl-O3, 20211021 5F (2) and 20211021 5R were utilized to amplify the open reading frame of cytoskeletal protein FtsZ, which was fused with green fluorescent protein mNoengreen (labelled as FtsZ_mNeongreen, or F-m) from the plasmid pZX9:FtsZ_mNeongreen [11]. Next, we used primers 20211021 6F and 20211021 6R to amplify the *Streptococcal* replicon and spectinomycin-resistant gene from the plasmid pZX9. These three linear fragments were subsequently ligated using Gibson assembly kit (Figure 1(d)), followed by transformation into *S. mutans* and selection on BHI plate with spectinomycin.

#### RNA extraction and real-time PCR

Total RNA was extracted from log-phase culture mixtures using an RNApure Bacteria kit (CWbiotech) according to the manufacturer’s instructions. The genes *egfp* for Xyl-S2, *mNeongreen* for Xyl-S1 and TC-Xyl and a control gene 16s RNA were selected to perform quantitative PCR (qPCR). The primers are listed in Table 2. One nanogram RNA was used to prepare the qPCR mixture with Hifair® III One Step RT-qPCR SYBR Green Kit (YEASEN, China) as the manual. The qPCR and results analysis was performed using the Quantagene q225 real-time PCR system as the manufacturer’s recommendations (Kubo Technology, China).

### Western blot analysis

An overnight culture grown in TH medium was diluted (1:00) in the 4 ml fresh TH medium with different xylose concentrations, and incubated to OD_600_ late log-phase (6 ~ 7 h). Cells were harvested at 3,000 × g for 3 min, washed once with 1 × phosphate-buffered saline (PBS) buffer, and resuspended in RIPA lysis buffer (Beyotime, China) supplemented with 1 mM PMSF (Thermo Fisher) and incubation for 1 h on ice. The whole cell proteins were boiled and separated by 10% sodium dodecyl sulfate polyacrylamide gel (SDS-PAGE) electrophoresis, transferred to polyvinylidene difluoride membranes (PVDF), blocked with 5% skim milk for 2 h at room temperature, and incubated with customed anti-FtsZ (1:5000, S-Evans Biosciences Ltd., Hangzhou, China) and anti-*S. mutans* (1:2000, ab31181, ABclonal) for 2 h at RT. After washing three times with tris-buffered saline plus Tween-20 (TBST), the membrane was incubated with secondary antibody (1:10000, BE0101, EASYBIO, China) for 1 h, followed by three times wash with TBST and incubation with ECL reagent. The images were collected with AI600 (GE).

### Sample preparation for fluorescent imaging

An overnight culture grown in TH medium was diluted (1:00) in the 1 ml fresh TH medium with or without xylose, and incubated to OD_600_ 0.4–0.5. One microliter bacterial culture was loaded between a gel pad (1× PBS supplemented with 1.5% low melting agarose) and coverslip (Fisher 24 × 50, No. 1.5). The fluorescent images were collected via N-STORM system (NIKON) equipped with 100 × oil objective. mNeongreeen was excited by 488 nm laser with a 100 ms exposure time. One hundred frames for each sample were collected, and the intensities were further averaged in ImageJ (NHI, Bethesda, MD, USA) to smooth the background noise.

### *Sample preparation for* △tsZ *strain*

An overnight culture grown in TH (1% xylose) medium was diluted (1:100) in fresh TH (1% xylose), and incubated to OD_600_ 0.4–0.5. The cultures were diluted to 10^−3^, 10^−5^, 10^−7^, and cultured on BHI plates (with or without xylose) for 48 hours. For the cell morphology imaging, 1 μl bacterial culture was loaded between a medium gel pad (TH supplemented with 1.5% low melting agarose) and coverslip. The cells were further cultured overnight at 37°C. The images were collected via the N-STORM system (NIKON) equipped with 100 × oil objective.

## Results

### TC-Xyl cassette shows undetectable basal expression

The binding of xylose operon repressor (XylR) to the *xylA* operator (xylAO), which is located immediately downstream of the gene promoter, influencing the gene transcription [10,12]. A previous study reported Xyl-S1 cassette employing the *ldh* promoter to achieve a high level of post-induction expression (Figure 1(a)) [10] but also showed a strong non-inducible expression. Hence, to accomplish tightly controlled and highly induced expression, we present the design of TC-Xyl, which is derived from the Xyl-S1 cassette. Compared to Xyl-S1, TC-Xyl includes two additional xylAO sequences downstream of the *ldh* promoter (Figure 1(a)). These two xylAo sequences were flanked by two 17 bp artificial sequences and incorporated into the Xyl-S1 cassette using Gibson assembly (Figure 1(b,c)). Following sequencing validation, the TC-Xyl cassette and the fragment containing the *Streptococcal* replicon and spectinomycin-resistant gene were cloned to produce plasmid pYL01 (Figure 1(d)). Concurrently, we cloned the open reading frame of the cytoskeletal protein FtsZ fused with the green fluorescent protein mNoengreen (FtsZ_mNeongreen) into pYL01 as the reporter.

**Figure 2.**
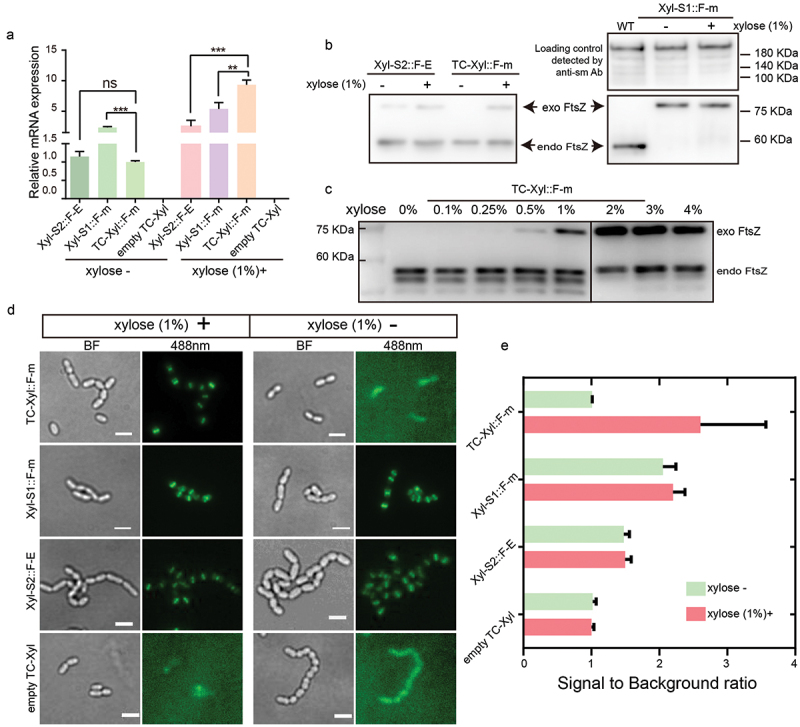
TC-Xyl shows tightly controlled gene-inducible expression. (a) the changes in relative mRNA expression of EGFP and mNeongreen in UA159 wild-type strains were investigated via real-time PCR. F-E: FtsZ_EGFP; F-m: FtsZ_mNeongreen. WT: wild type. ‘***’ *p* value < 0.001; ‘**’ *p* value < 0.01. ‘ns’ no significance. The *p* value was determined via student’s t-test. (b) the protein expression levels of exogenous FtsZ (exo FtsZ) under the control of TC-Xyl, Xyl-S1, and Xyl-S2 in UA159 wild-type strains were examined by Western blot. Endogenous FtsZ (endo FtsZ) was used as reference. For group of Xyl-S1, the whole cell proteins were set as reference and detected by anti-sm. (c) the expression of FtsZ_mNeongreen under the control of TC-Xyl in the presence of different concentration xylose were analyzed by Western blot. (d) the expression of FtsZ_mNeongreen in WT was analyzed by the fluorescent microscope. The cell morphology pictures were collected via bright field illumination. mNeongreen was excited by a 488 nm laser. Scale bar: 2 μm. (e) the signal to background ratios before and after induction were analyzed. The mean with SD were shown.

Next, to assess whether TC-Xyl offers stringent gene repression and high inducible gene expression capacity, we first measured the mRNA expression of FtsZ-fused fluorescent proteins mNeongreen and EGFP via Real-time PCR in *S. mutans* UA159 wild-type strain. We observed a wider range of induced expression in TC-Xyl. In the absence of inducer, the basal expression of TC-Xyl could be observed, similar to that of Xyl-S2. With 1% xylose induction, gene expression was higher than that of Xyl-S1. We also examined the mNeongreen transcripts in the strain containing an empty TC-Xyl plasmid, which lacks the reporter FtsZ_mNeongreen, confirming these transcripts were not due to an unspecific reaction (Figure 2(a)). Although basal gene expression of TC-Xyl could be detected at the mRNA level, the results of the Western bolt demonstrated that the basal expression of FtsZ_mNeongreen was not detected at the protein level in the absence of xylose. In contrast, the basal expression of the exogenous FtsZ_FP under the control of Xyl-S1 and Xyl-S2 could be easily detected irrespective of the presence or absence of xylose (Figure 2(b)). Unexpectedly, the exogenous expression of FtsZ_mNeongreen under the control of the Xyl-S1 cassette inhibited the expression of endogenous FtsZ. One plausible explanation is that the basal expression level of FtsZ_mNeongreen in Xyl-S1 equals to that of the endogenous FtsZ, prompting the host strain to use the exogenous FtsZ_mNeongreen as the sole source to ensure physiological stability. Thus, to avoid the influence of the loading volume on the results of Xyl-S1, we used whole cell proteins of *S. mutans* as the loading control, confirming its high basal expression level.

Next, to illustrate how the expression level of FtsZ_mNeongreen was affected by the inducing compound xylose concentration, we added 0.1%, 0.25%, 0.5%, 1%, 2%, 3%, and 4% xylose to the medium. The result showed that the expression of FtsZ_mNeongreen was detected only after the xylose concentration reached 0.5%, indicating an extremely low basal expression level of the TC-Xyl cassette. When the concentration reached 1%, the exogenous FtsZ_mNeongreen demonstrated a similar expression level to the endogenous FtsZ. The expression level peaked and did not further increase with rising xylose concentration once the concentration of xylose reached 2% (Figure 2(c)).

Next, we investigated the expression and localization of FtsZ_mNeongreen using a fluorescence microscope. We found that the TC-Xyl group displayed an obvious fluorescence signal and demonstrated a typical middle localization of FtsZ, when the medium was supplemented with 1% xylose. However, when the strain was incubated in the medium without xylose, no signal of FtsZ-mNeongreen could be detected (Figure 2(d)), suggesting a tight gene expression control of TC-Xyl. Meanwhile, the strain containing Xyl-S1 and Xyl-S2 consistently showed an obvious signal of exogenous Fts, regardless of the presence or absence of xylose in the medium, confirming their high basal expression. To quantitatively illustrate the difference in fluorescence signal of FtsZ_mNeongreen before and after induction, we evaluated the ‘signal-to-background ratio (SBR)’. We found that without the xylose, the SBR values of the TC-Xyl group were close to 1 and similar to that of the control group empty TC-Xyl and significantly lower than that of the Xyl-S1 and S2 groups. Further, after the addition of xylose, a significant increase in SBR of TC-Xyl could be detected (Figure 2(e)). Altogether, the results of the molecular method and microscopy-based technique demonstrated an extremely tight control of gene expression of the TC-Xyl cassette.

### Depletion of FtsZ lead to cell morphology change and cell death

**Figure 3.**
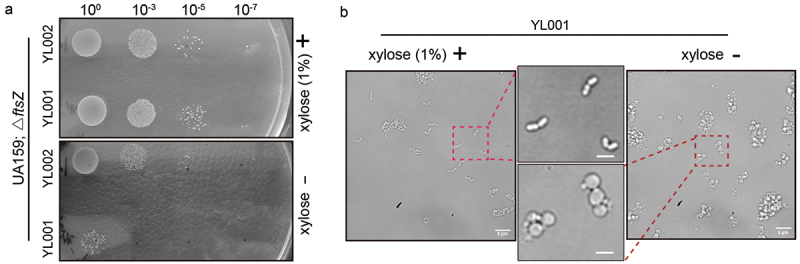
Growth phenotypes of FtsZ depletion in *S. mutan*s. (a) Growth of different dilutions of strain YL001 and YL002 on BHI plate with or without xylose. (b) The cell morphology change after FtsZ depletion were analyzed by live cell microscope. Red dot line box marked the enlarged area. Scale bar: 2 μm.

Next, in order to illustrate the advantages of the TC-Xyl cassette for studying the function of lethal genes, we investigated the function of the cytoskeletal protein FtsZ. FtsZ is a core component of cell divisome for almost all bacteria [13]. Studying its function in *S. mutans* has been challenging due to difficulties in culturing gene knock out mutations. In this study, we knocked out the endogenous *ftsZ* via an in-frame deletion [14] while simultaneously expressing an exogenous copy of the *ftsZ_mNeongreen* under the control of TC-Xyl (strain YL001) and Xyl-S1 (strain YL002) as the sole source of FtsZ. After overnight culture, the YL001 and YL002 in BHI with 1% xylose, we diluted it on BHI plates with or without xylose. We observed that mutation YL001 grew without apparent defects when the medium was supplemented with 1% xylose, but the number of surviving colonies dramatically decreased in the absence of xylose. This confirms the lethal role of FtsZ in *S. mutans* (Figure 3(a)). Unlike strain YL001, a growth defect in YL002 on the plate without xylose was only observed when the mixtures were diluted beyond to 10^−3^.

*S. mutans* typically displays a regular slightly elongated cell shape under normal conditions. Interestingly, using live cell imaging, we found that depletion of FtsZ resulted in a round-like cell shape, exhibiting a wider width and mini cell formation (Figure 3(b)). However, the strain maintained a normal cell shape when the culture medium was supplemented with 1% xylose, suggesting the essential role of FtsZ in maintaining cell shape stability and cell division. Thus, we have shown that TC-Xyl can serve as a powerful tool for the functional study of lethal genes.

## Discussion

A tightly controlled gene induction expression system can significantly contribute to the study of gene function in *S. mutans*. Existing systems in *S. mutans* either display high basal expression levels [7–10] or low inducible expression levels [6] in *S. mutans*. In this study, we introduce a tightly controlled xylose-inducible gene expression system, TC-Xyl. This system incorporates three repetitive xylAo fragments downstream of the *ldh* promoter (Figure 1), which provide more binding sites for XylR resulting in a decrease in the uninduced gene expression. The results of the molecular methods and microscopy-based technique demonstrate that TC-Xyl exhibits an extremely low uninduced expression level (Figure 2), which has important implications for the study of lethal gene function.

FtsZ is an essential gene involved in the cell division process in almost all bacteria [13]. The function of FtsZ is considered to be to form a ring-like structure during the early stage of division, facilitating septum formation [13]. Thus, to showcase the application of TC-Xyl in the lethal gene functional studies, we knock out the native *ftsz* ORF while simultaneously expressing the FtsZ_mNeongreeen under the control of TC-Xyl as the sole source. The results of the growth phenotype on the plate confirmed the essential role of FtsZ in *S. mutans* (Figure 3(a)). Notably, the growth defect of YL001 on the BHI plate without xylose was easily observable even in the undiluted culture mixture, indicating the strong repression of *ftsZ* transcription, which was significantly better than that of previously reported Xyl-S2 cassette [10,15].

In the rod-shaped model organism *Escherichia coli*, FtsZ dysfunction results in the septum disappearing and alters the cell shape into an elongated filament-like cell [16,17]. However, we observed that the cell shape in *S. mutans* changed from a regular cell shape to an enlarged spherical cell shape due to FtsZ depletion (Figure 3(b)), which is similar to another ovoid-shaped pathogen *Streptococcus pneumoniae* [18]. One plausible explanation could be that during cell division, chromatin replication results in more cellular contents. However, the cell is unable to divide, ultimately causing the cell to expand.

Given that xylose is not metabolized and does not exhibit apparent toxicity to *S. mutans*, we believe that TC-Xyl can be applied to all gene induction studies in *S. mutans*, particularly sugar metabolism-related studies and the titration of lethal gene expression. Recently, CRISPR-based techniques like CRISPRi [19] (gene transcription interference), CRISPRa [20] (gene transcription activation), and CRISPR-Csm-based RNAi [21] have provided powerful platforms for achieving large-scale gene function studies. The combination of TC-Xyl with these technologies can substantially facilitate our in-depth study of molecular mechanisms in *Streptococcus mutans*.
